# Causal factors for seismicity near Azle, Texas

**DOI:** 10.1038/ncomms7728

**Published:** 2015-04-21

**Authors:** Matthew J. Hornbach, Heather R. DeShon, William L. Ellsworth, Brian W. Stump, Chris Hayward, Cliff Frohlich, Harrison R. Oldham, Jon E. Olson, M. Beatrice Magnani, Casey Brokaw, James H. Luetgert

**Affiliations:** 1 Huffington Department of Earth Sciences, Southern Methodist University, Dallas, Texas 75275, USA; 2 USA Geological Survey, 345 Middlefield Road, MS977, Menlo Park, California 94025, USA; 3 Institute for Geophysics, The University of Texas at Austin, Austin, Texas 78758-4445, USA; 4 Department of Petroleum and Geosystems Engineering, The University of Texas at Austin, Austin, Texas 78712-2100, USA

## Abstract

In November 2013, a series of earthquakes began along a mapped ancient fault system near Azle, Texas. Here we assess whether it is plausible that human activity caused these earthquakes. Analysis of both lake and groundwater variations near Azle shows that no significant stress changes were associated with the shallow water table before or during the earthquake sequence. In contrast, pore-pressure models demonstrate that a combination of brine production and wastewater injection near the fault generated subsurface pressures sufficient to induce earthquakes on near-critically stressed faults. On the basis of modelling results and the absence of historical earthquakes near Azle, brine production combined with wastewater disposal represent the most likely cause of recent seismicity near Azle. For assessing the earthquake cause, our research underscores the necessity of monitoring subsurface wastewater formation pressures and monitoring earthquakes having magnitudes of ∼M2 and greater. Currently, monitoring at these levels is not standard across Texas or the United States.

## Abstract (web summary)

Whether exploration causes earthquakes has been a matter of recent contention particularly regarding shale gas exploration. Here, the authors use hydraulic modelling and earthquake locations to show that brine production and wastewater injection in the Azle area are likely causing earthquakes.

## Graphical abstract

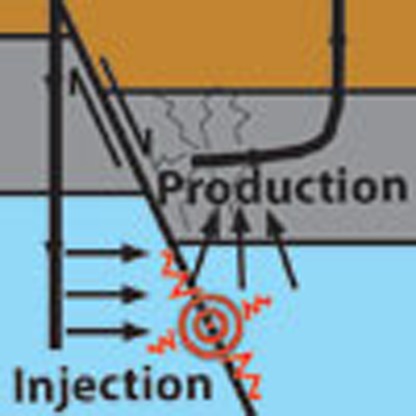

Several factors, both natural and anthropogenic, can reactivate faults and cause earthquakes^1^,^2^,^3^,^4^. These factors include, but are not limited to, stress changes caused by the natural shift of Earth's plates, stress changes induced by water table fluctuations^2^,^3^ and stress changes induced by the removal and the injection of fluids in the deep subsurface^4^ (Fig. 1). We use the term ‘induced' to include earthquakes triggered by anthropogenic causes that release tectonic stress as well as earthquakes that release stresses created by industrial activity^1^. Determining which factor is the primary driver of seismicity is often difficult without a detailed understanding of the subsurface stress regime and geology.

Surveys of crustal stress and observations from deep boreholes at locations worldwide indicate that stress in continental interiors maintains consistent orientation within the regional provinces having dimensions of hundreds to thousands of km^5^; the brittle crust is often in a state of near-failure equilibrium^6^; although aseismic deformation can occur, stress levels are often limited by the frictional strength of pervasive naturally occurring faults governed by Coulomb frictional failure theory^7^; and increased fluid pressure along faults promotes failure by reducing effective stress^8^. In areas where the Earth's crust is critically stressed, surprisingly small changes in stress (typically 0.01–0.1 MPa) can trigger fault reactivation and cause earthquakes^9^,^10^.

Both nationally^1^,^11^,^12^,^13^,^14^,^15^ and in Texas^16^,^17^,^18^,^19^, studies examining the recent seismicity in oil- and gas-producing areas often attribute earthquakes to high-volume wastewater injection based on the proximity of injection wells to hypocenters and because the onset of seismic activity follows the emplacement and use of injection wells. Most of these studies, however, do not evaluate other possible anthropogenic causes of seismicity or do not utilize physical models to quantify stress change. Critics of these studies note, correctly, that tens of thousands of currently active injection wells apparently do not induce earthquakes or at least not earthquakes large enough to be felt or recorded by seismic networks^4^. Why some injection wells induce seismicity while others do not is unclear. Here we consider several regional factors that might cause seismicity near Azle, Texas.

This analysis demonstrates that brine production combined with wastewater injection generates more significant subsurface stress changes at earthquake depths than regional groundwater or lake level changes. Regional geologic interpretations and historical accounts of regional seismicity independently suggest that natural tectonic stress changes represent an unlikely cause of the Azle earthquakes. The analysis therefore indicates subsurface stress changes associated with brine production and wastewater injection represents the most probable cause of recent earthquakes in the Azle area. The study highlights the need for better subsurface pore pressure and seismic monitoring to address future potential-induced seismicity hazards.

## Results

### Linking seismicity with regional geology

**Figure 2:**
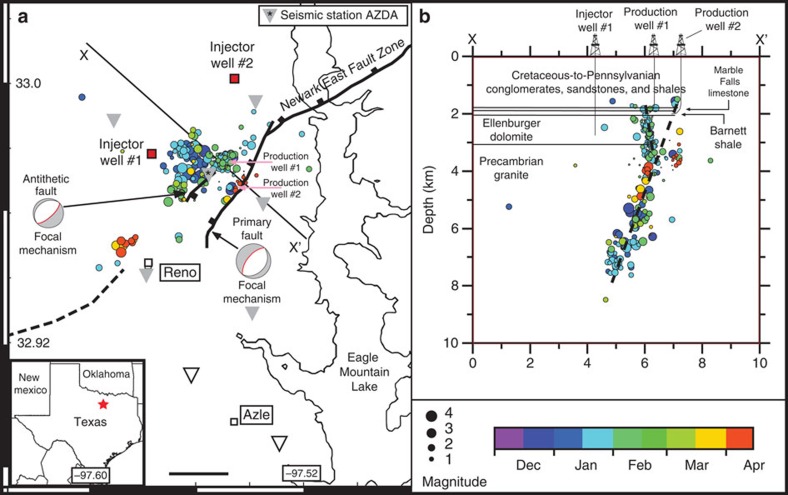
Azle Earthquake locations and regional geologic structure. Map showing the location of NEFZ (black) at the top of the Ellenburger formation, inferred faults (dashed) at the top of the Ellenburger formation, injection wells (red squares), two production wells (API 36734045 and 36734139) with significant brine production near the faults (pink arrows) and earthquake epicentres (coloured circles) recorded by the temporary seismic network (triangles) (**a**). The red star in the inset of **a** shows the map location. The black scale bar in **a** is 2 km. Grey (white) triangles indicate the locations of active (inactive) seismic stations. Line X–X′ in **a** shows the location of the cross-section shown in (**b**). We interpret two faults based on earthquake location and consistent with industry interpretations: a primary normal fault and a shallower antithetic normal fault.

**Figure 3:**
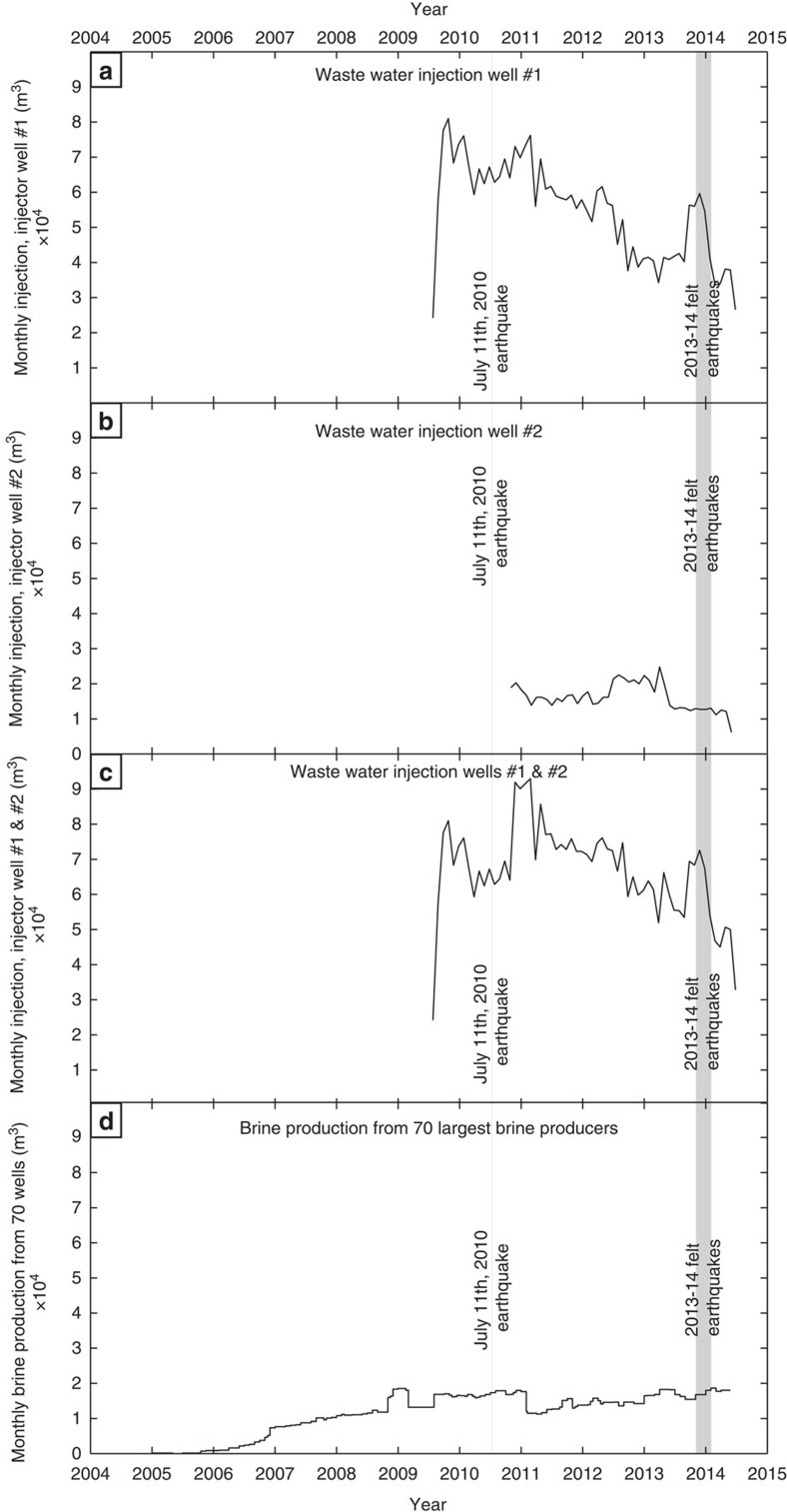
Regional wastewater injection and production volumes versus time. Monthly injection volume versus time at injector well #1 (**a**). Monthly injection volume versus time at injector well #2 (**b**). Combined monthly injection volume for both injector well #1 and injector well #2 (**c**). Estimated monthly water production with time for the 70 largest water producing wells within 10 km of earthquake epicentres (**d**). Note that the scales for all of these plots are the same.

From early November 2013 through January 2014, the United States Geological Survey's National Earthquake Information Center (NEIC) reported 27 earthquakes near the cities of Azle and Reno, Texas, including two widely felt M3.6 events (Fig. 2 and Supplementary Fig. 1). To improve locations, refine magnitudes and characterize the fault geometry associated with the events, a temporary local seismic network was deployed in mid-December 2013 (see Supplementary Fig. 1; Supplementary Table 1). We report high-accuracy earthquake locations and magnitudes based on these data for earthquakes occurring up through 30 April 2014 (Figs 2b and 3). Seismicity occurs on two steeply dipping, conjugate faults consistent with the general strike of the Newark East fault zone (NEFZ)^20^ (Fig. 2a,b). First motion composite focal mechanism solutions are consistent with a primary normal fault extending down-dip through the crystalline basement (strike 225°, dip ∼60–70°) and a more steeply dipping (∼70–80°) shallow conjugate normal fault (Fig. 2b). Earthquake locations using regional velocity models (Supplementary Table 2) suggest that both faults extend into the overlying Ellenburger sedimentary unit, and formation depths based on well logs indicate perhaps 100 m of offset exists along the primary fault^20^, with the fault dipping to the northwest. Earthquake focal mechanisms and fault orientations are consistent with previous stress regime studies suggesting that the maximum principal stress is vertical in this area^5^,^6^. On the basis of the conversations with industry representatives, the location and dip of the faults defined in our three-dimensional (3D) fault model using passive source seismic data are consistent with industry regional fault interpretations using 3D seismic data.

The Newark East Gas Field (NEGF), a major gas-producing field in the Fort Worth Basin, extends north and east of Azle^20^. Hydraulic fracturing is applied to produce gas from the low permeability (∼10^−18^–10^−19^ m^2^) Mississippian Barnett Shale (Fig. 2b). Along with natural gas, hydraulically fractured gas wells in the Azle area of the NEGF can unintentionally produce (and remove from the subsurface) significant volumes of water, mostly brine, through fractures that extend to the underlying high-permeability (10^−14^–10^−15^ m^2^) Ellenburger formation, a flat-lying ∼1,000-m thick dolomitic limestone^20^,^21^. Brine and fracturing fluid produced from production wells are reinjected through disposal wells completed in the Ellenburger formation. Lower permeability (10^−19^–10^−20^ m^2^) Precambrian granite underlies the Ellenburger^20^ (Fig. 2b).

At least one major (>50 km long) fault system, the NEFZ, extends northeast–southwest across the NEGF where recent Azle earthquakes occurred. Comparing the earthquake locations with multiple structural interpretations provided by industry representatives, it appears that the deeper earthquakes occur along part of the main NEFZ, whereas many shallow earthquakes associated with short-duration seismic swarms occur along a conjugate fault likely associated with a collapsed Ellenburger karst feature^21^,^22^ (Fig. 2). The location and geometry of this fault system is complex and difficult to constrain in the area of recent earthquake activity but is well defined to the northeast^20^ (Fig. 2). On the basis of discussions with industry concerning proprietary seismic data, the fault is well imaged through the production and injection depth intervals. Above the production interval and within the Precambrian basement, the fault geometry is not well known. Historically, there has been no evidence for seismicity in this region or along this fault^23^.

### Assessing water table stress changes

Induced seismicity is sometimes attributed to water-level and lake-level variations^2^,^3^. Eagle Mountain Lake is a large reservoir located ∼5 km east of the earthquake epicentres; drought caused it to drop in elevation by 2.1 m from April 2012 to November 2013. Our calculations indicate that at Ellenburger depths, this water level drop will reduce Coulomb stress by only ∼0.0006 MPa (Supplementary Fig. 2). This is one to three orders of magnitude smaller than typical stress changes associated with triggered seismicity^9^,^10^, although KPa stress changes do sometimes trigger earthquakes at other locations^24^. Eagle Mountain Lake water level changes during the past few years, however, are within historic values (Supplementary Fig. 3). It is therefore difficult to attribute recent seismicity in Azle to lake level change.

We also looked at variations in the Trinity Aquifer recorded at groundwater monitoring wells (Supplementary Fig. 4). The unconfined Trinity Aquifer exists ∼100 m below the surface near Azle. Although data are limited, where data exist we observe no significant or systematic changes in the depth of the aquifer for the past 6–8 years. This implies water level changes in the aquifer are not responsible for recent seismicity.

### Assessing natural tectonic stress changes

Though rare, natural intraplate tectonic stress changes have reactivated ancient fault systems far from known seismic zones, causing earthquakes^25^,^26^. We acknowledge that it is possible, but unlikely, that natural tectonic stress changes are responsible for recent seismicity in Azle since the region is historically inactive. Before the occurrence of probable injection-induced earthquakes in the Dallas-Fort Worth area in 2008, the historic seismicity record includes only one felt earthquake within the 140,000 km^2^ Fort Worth Basin^16^,^17^. That felt event, however, is based on a single felt report with no associated stories in regional newspapers and suspected poor location quality. One small (<M2.5), unfelt earthquake (11 July 2010) was detected in the Azle region when the Earthscope Transportable Array moved through North Texas^17^. The unusual increase in north Texas seismicity since 2008 is consistent with other seismicity studies in the central United States that document significant increases in the rate of earthquakes greater than magnitude 3.0 in the past 6 years^1^,^13^,^14^,^15^,^16^,^17^,^18^,^19^. These studies generally conclude that the recent increase in US seismicity is not a natural phenomenon but is instead caused by human practices, primarily wastewater injection.

No obvious surface expression exists for the NEFZ, implying no recent surface rupture, and although only limited publically available seismic data exist, analysis suggests that the majority of faults extending through the Ellenburger are associated with karst collapses that occurred ∼300 Myr ago^21^,^22^. This is consistent with the observation that until 2010, no earthquakes had been either recorded or felt in the Azle region during more than 150 years of settlement^16^,^17^,^23^. On the basis of earthquake locations, focal mechanisms and regional seismic interpretations, most shallow Azle earthquake events occur along the antithetic fault associated with a ∼300 million-year-old Ellenburger karst collapse. Although long-term stress monitoring is ultimately needed, the lack of evidence for significant faulting in the region during the last ∼300 million years and the fact that no reliable historic earthquake reports exist near Azle during the past ∼150 years of permanent settlement supports the premise that natural intraplate tectonic stress changes are an unlikely cause of seismicity in this region.

### Assessing stress changes associated with oil and gas activity

Several production and injection wells drilled during the past decade in the Fort Worth Basin represent an additional potential cause of seismicity^4^. Two high-volume wastewater injection wells (Fig. 2a) and more than 70 production wells that produce gas and brine (Supplementary Fig. 5) are situated within 10 km of the Azle earthquake sequence and the NEFZ. Average monthly wastewater injection pressures and volumes are available from the Texas Railroad Commission (TRC). injector well #1 began injecting in June 2009 and has averaged ∼44,000 m^3^ per month (Fig. 3a, Supplementary Fig. 6). Injector well #2 began injecting in October 2010 and has averaged ∼13,000 m^3^ per month (Fig. 3b, Supplementary Fig. 6). Injection pressures are reported only at the wellhead, and the TRC collects no downhole formation pressures or subsurface shut-in pressures that would allow for formation pressure monitoring. Fluid production from oil and gas production wells in this region, including brine likely from the Ellenburger, is only reported to the TRC during the annual 48-h pump tests (G-1 and G-10 forms) and is highly variable—typically ranging from 0 to 800 m^3^ per month per well. Since G-10 reporting typically occurs only on an annual basis, it provides only a crude estimate of brine production across the region. We use G-10 production reports combined with gas production reports for the 70 largest brine-producing wells in the region to make first-order estimates of brine production (Fig. 3d). In general, the most significant brine production occurs along the NEFZ.

It is difficult to draw a simple correlation between the timing of fluid injection, fluid production and seismicity in Azle (Fig. 3). Although there is an increase in injection volumes in mid-2013 before the recent events (Fig. 3a), even higher volumes and pressures are reported in prior years at both injectors, when no felt earthquakes occurred (Fig. 3, Supplementary Figs 6 and 7). A key issue is how fluid pressure changes caused by the injection and removal of fluid impact subsurface stress along the fault. To estimate how fluid pressure changed over time and space in the area of earthquake activity, we developed a 3D pore pressure model for the Ellenburger formation. The model calculates variations in subsurface pressure due to two regional wastewater injection wells and the 70 largest brine production wells in the modelling domain located near NEFZ earthquake activity^27^.

We ran the model for a 10-year period from 2004 to the end of 2013 over a range of parameters (see, for example, Table 1). Permeability in the Ellenburger is constrained using pump test data supplied by energy companies (Supplementary Fig. 8). Injection and production well pressures are varied with time based on data provided by the TRC (Supplementary Tables 3–5). We begin the model run in 2004 to account for the 70 regional brine production wells that may have removed water from the Ellenburger as early as 2004, thereby reducing the pressure. We vary brine production monthly so that it only occurs when a well is also producing gas. Owing to uncertainties in gas production and gas volumes in the Ellenburger, the model currently does not account for multiphase flow.

**Figure 4:**
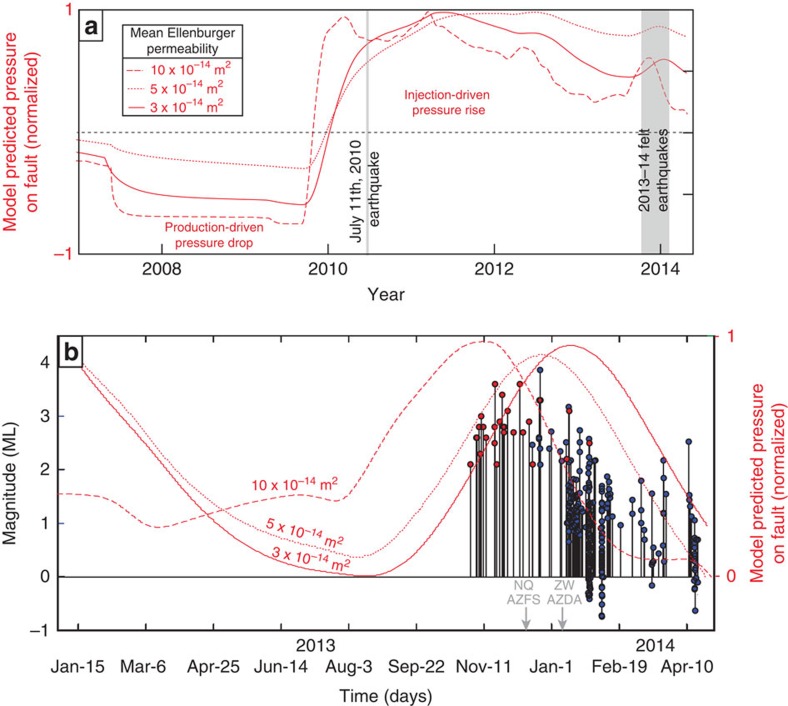
Pressure at the antithetic fault versus time. Modelled pressure versus time at the antithetic fault, directly below seismometer AZDA (Fig. 2a) (**a**). Results include three different mean Ellenburger permeability values and demonstrate earthquake activity correlates in time with a local pressure maximum but not an absolute maximum at this site. Higher resolution time image of modelled injection pressures versus time at AZDA with earthquakes (stem and circle) coloured by network (NEIC-red; SMU-blue) (**b**). In 2010, one small (<M 2.5) earthquake was detected in the study area^17^. Event detection increases beginning on 15 December, the date when the first Netquakes station (NQ_AZFS) was deployed. Detection further improved when station ZW_AZDA was installed. Model results indicating pressures increase along the fault near the time of felt seismicity, with a 1–3-month delay between injection rate increase and pore pressure change at the fault based on permeability values measured at injector well #1.

**Figure 5:**
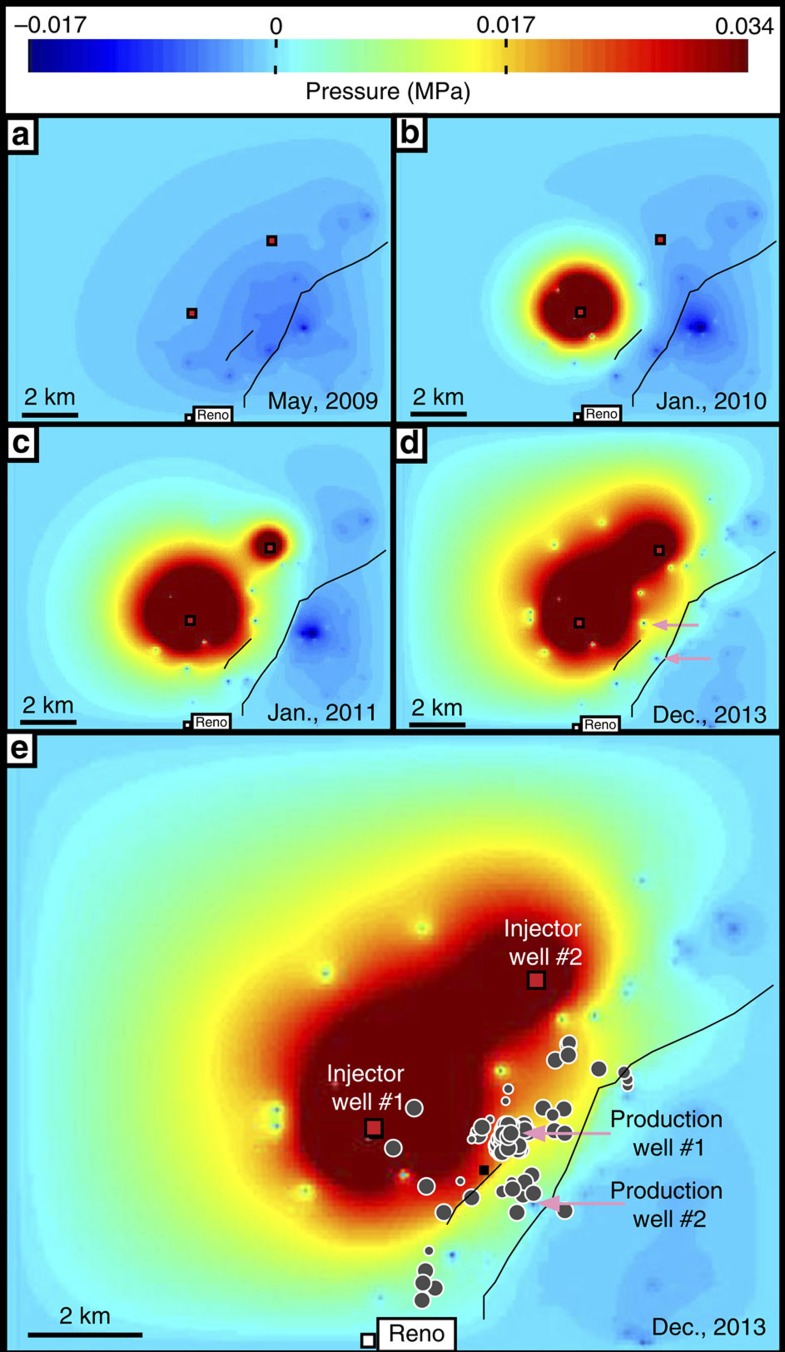
Modelled pressure changes in the Ellenburger caused by injection and production. Map view of modelled excess pressures at a depth of ∼2,500 m for May 2009 (**a**), January 2010 (**b**), January 2011 (**c**) and December 2013 (**d**,**e**). The model uses average monthly reported water injection rates and the Dupuit–Theim equation to estimate bottom-hole pressure values. Pressure above hydrostatic averages 0.58 MPa for injector well #1 and 0.28 MPa for injector well #2 during injection. Ellenburger permeability is assumed constant at 5 × 10^−14^ m^2^; boundary conditions are open along the side and closed at the top and bottom. We apply an average rate of brine production based directly on reported TRC G-10 water production values for the 70 largest water producing production wells in the region. The images show the system before injection (**a**) through the onset of seismicity (**e**). Black lines, the NEFZ location at the top of the Ellenburger formation; red squares, injector locations; pink arrows, approximate location of two large brine production wells that are located both near the faults and near reported earthquakes swarms within the Ellenburger (grey circles with white outlines). Note that the most significant amount of brine removal occurs along the fault trend (**a**).

Model results show that a pressure differential develops along the antithetic fault as a combined result of high fluid injection rates to the west and high water removal rates to the east (Figs 4 and 5). While the absolute pressure change depends on input parameters (Table 1), in the area of recent seismicity, the differential pressure development along the faults remains a consistent feature of all model runs. Modelled pressure changes on the faults typically range between 0.01 and 0.2 MPa, depending on model parameters (see, for example, Table 1). Although uncertainty exists, the model-predicted pressure changes are consistent with values that are known to trigger earthquakes on critically stressed faults^9^,^10^ and are one to three orders of magnitude greater than stress changes associated with lake and water table changes in the region. This is true even when we use end-member bottom-hole injection pressures that are an order of magnitude lower than reported wellhead injection pressures (see, for example, Table 1, Supplementary Fig. 9).

## Discussion

It is notable that we observe earthquake swarms in the Ellenburger apparently associated with extraction, not just injection, that is, they occur almost directly below the estimated subsurface location of two large brine production wells in the region, as indicated by TRC G-10 reports. On the basis of fault and well locations and the nature of permeability along faults, it is likely that these two production wells remove fluids from sediments immediately adjacent to the fault^7^,^28^. Earthquakes caused by fluid extraction near faults are not a new phenomenon in the United States or even Texas^29^,^30^,^31^. Induced seismicity is often associated with subsurface pressure changes, and extensional stresses will concentrate on the boundary of the fluid draw-down region, promoting normal faulting^29^,^32^. It is therefore perhaps no coincidence that we observe swarms of normal-faulting events in regions where more significant near-fault stress changes occur (Fig. 5d,e).

For simplicity, the model assesses pressure only in the Ellenburger formation where several earthquakes were recorded. The absolute focal depth of several of the initial large NEIC catalogue events remains unknown, but the larger magnitude earthquakes recorded by the temporary network occur in the underlying basement along the primary fault (Fig. 2a,b). We hypothesize that the deeper earthquakes are due to downward pressure transfer within the fault system. If the underlying basement granite has very low (<10^−19^ m^2^) permeability, pressure transfer will preferentially occur along the higher permeability fracture zone and damaged zones within and parallel to the fault^7^,^28^. Little is known about the permeability along the unconformity between the Ellenburger and granite basement. Currently, no publically available permeability data exist for either basement rock or the NEFZ, making it difficult to assess pore pressure change along the fault system below the Ellenburger. Industry researchers have, however, drilled through the NEFZ in the Barnett, and they suggest regional permeability is complex, with both high- and low-permeability pathways associated with the fault, consistent with detailed permeability studies of faulted formations^28^.

Modelling results indicate that a combination of formation fluid production and wastewater injection generate the most significant stress changes at earthquake depths compared with other studied phenomena (such as groundwater or lake level fluctuations). The lack of evidence for both regional fault reactivation during the past ∼300 million years and regional seismicity for the past ∼150 years also supports the conclusion that brine production and wastewater injection represent the most likely cause of recent seismicity near Azle. The location, magnitude and timing of oil- and gas-generated subsurface pressure changes provide a more consistent explanation for recent seismicity than the other causal factors analysed. A complex interplay between brine production and wastewater injection likely promotes seismic activity. Nonetheless, several uncertainties remain, in part, due to the limited amount of data available that would allow more accurate calculations of *in situ* stress and possible changes in the subsurface stress regime over time (Table 1).

Nearly 50 years ago, industry researchers such as Van Everdingen^33^ recognized the critical importance of baseline monitoring of subsurface pressures and fluid volumes in wastewater reservoirs to minimize hazards. Baseline pressure monitoring data, including shut-in pressure tests and pump-tests are easy to obtain, routinely collected by industry and invaluable in assessing reservoir permeability and subsurface pressure changes with time, but are currently neither required nor typically available for analysis. Similarly, improved regional seismic monitoring in areas of ongoing or potential oil and gas activity can provide invaluable insights concerning areas of potential seismic hazards. To address fully the role oil and gas activities play in promoting earthquakes, and to prepare properly for the future, induced earthquake hazard analysis ultimately requires significantly more comprehensive data sets than are currently available. These data should accurately monitor and quantify not only seismicity, volume changes and subsurface stress, but also regional subsurface structure and stress changes in space and time within a well-constrained 3D geologic framework.

## Methods

### Earthquake locations

Twenty-seven felt earthquakes were reported by the USGS NEIC through 28 January 2014 (Supplementary Fig. 1). Owing to the sparse distribution of seismic stations, routine NEIC earthquake location uncertainty in North Texas is roughly 10 km, and the initial locations exhibited a spread of nearly 20 km west to east. To reduce the location uncertainty and characterize the size and faulting associated with the earthquakes, we deployed five temporary seismic stations in the Reno–Azle area in mid-December 2013 and completed a 12-station deployment in January 2014 (Supplementary Table 1). Stations are a mix of USGS NetQuakes accelerometers, broadband and short-period velocity sensors, and one infrasound sensor recording at 100 or 200 Hz (Supplementary Fig. 1 and Supplementary Table 1). Waveforms from the USGS NetQuakes stations and SMU temporary stations, reported to the IRIS Data Management Center as network code NQ and ZW, respectively, for 2013/2014, are combined with other regional US seismic stations to provide consistent detection across multiple stations to a magnitude of ∼1.0.

All waveform data are telemetered in near real-time. P-wave onsets are identified using short-term-average/long-term average automated techniques applied to all vertical channels. Events are then manually reviewed for additional P- and S-wave onsets. Pick uncertainty is estimated to be 0.02 s for P-waves and 0.04 s for S-waves based on the sampling rates of the waveform data. Events are relocated using GENLOC, a flexible implementation of the Gauss–Newton inversion method applied to a single-event location^34^ and a layered one-dimensional (1D) velocity model developed for the Azle area (Supplementary Table 2). The 1D P-wave model is based on sonic log information from injection well #2 and published 1D models for other earthquake sequences in the Dallas/Fort Worth area^18^. Constant *V*_P_/*V*_S_ of 1.80 is applied to derive S-wave velocity^18^. Location uncertainty is reported as 68% confidence ellipses based on the formal covariance matrix for each earthquake^34^. For the 283 events reported here, the mean epicentre major axes length is 570±362 m, minor axes length is 310±228 m, depth uncertainty is 346±171 m and origin time uncertainty is 0.054±0.031 s. Tests using alternative 1D constant *V*_P_/*V*_S_, which may affect depth estimates outside of formal uncertainty ranges, results in mean depth changes of 450±820 m (deeper) for *V*_P_/*V*_S_ 1.732 and −650±1,400 m (shallower) for *V*_P_/*V*_S_ 1.90. The mean of the travel time residuals root mean square is 0.08±0.12 s.

The two composite focal mechanisms discussed in the main text were calculated by hand using P-wave first motions. The composite mechanism for the main fault used larger, well-recorded events from December 2014; the antithetic composite used larger events occurring on 28 January 2014. The mechanisms confirm that first motions were consistent with the overall strike, dip and normal faulting offset expected from 3D seismic data and hypocentres. Future work will focus on calculating a more comprehensive set of focal mechanism solutions using P, S and amplitude information.

Local (Richter) magnitudes are based on the maximum S-wave amplitudes recorded on the horizontal velocity and acceleration seismograms transferred to the Wood–Anderson displacement. Magnitudes are generally consistent with NEIC magnitudes for like events (Fig. 2d). Before the installation of station AZDA, magnitude completeness is estimated to be 1.6 ml; after AZDA, completeness increases to 1.0 ml and many smaller events down to −1.0 ml are accurately recorded during periods of swarm activity. Estimates of *b* value using the NEIC magnitudes and using the local magnitudes are ∼1.0. The initial catalogue locations and magnitudes reported are not a complete record of seismicity recorded on the temporary network but provide sufficient information to constrain fault geometry for modelling. Future work will refine locations, including periods of swarm activity and provide refined magnitude estimates with more accurate *b* value calculations.

### Modelling the effects of water level change

It has long been known that impoundment of artificial reservoirs can induce earthquakes either by the direct effect of the added surface load or the indirect effect of pore pressure diffusion to the earthquake focal region^2^. The close proximity of Eagle Mountain Lake to the Azle area earthquakes raises the possibility that the reservoir was involved in inducing the sequence. The west fork of the Trinity River was dammed in 1932 to form the lake with a maximum depth of 14 m. No felt earthquakes were reported in the vicinity of the reservoir for 150 years before October 2013 (refs 16, 17, 23). The hydrograph for the lake level (http://nwis.waterdata.usgs.gov/tx/nwis/uv/?cb_00054=on&cb_00062=on&format=gif_default&period=&begin_date=2007-10-01&end_date=2014-02-26&site_no=08045000) shows that over the past 2 years the lake level has declined by 2.1 m from the Conservation Pool Elevation of 198 m (Supplementary Fig. 3). A falling lake level can sometimes strengthen the faults that are in hydrologic connection with the pool since lower water pressure results in a higher effective stress, increasing your mean stress and moving the stress state away from the failure envelope. The changing reservoir load could, however, encourage failure and can be modelled using the Boussinesq solution for a change in load on the surface of an elastic half-space (Supplementary Fig. 2)^35^. The change in Coulomb stress created by the 2.1 m decline in lake level was computed on receiver faults corresponding to the main and antithetic faults imaged by the earthquake hypocenters at depths of 3 and 5 km (Supplementary Fig. 4). In the hypocentre zone, the Coulomb stress change is <1 KPa and of about the same size as tidal stresses. Because the change is likely one to three orders of magnitude smaller than the pore-pressure effect of injection on Coulomb stress we conclude that changes in the level of Eagle Mountain Lake can be ruled out as an important contributing factor to the Azle earthquake sequence.

### Physical modelling of subsurface pressures

To determine the location and approximate magnitude of subsurface pressures generated by the injection and production wells, we develop a 3D pore-pressure model for the Ellenburger formation near the area of recent earthquake activity. The fault and surrounding formations above and below the Ellenburger are treated as low permeability (10^−16^–10^−18^ m^2^) zones. We apply both open and closed boundary conditions for an assortment of runs (see Table 1).

The model incorporates both brine injection and brine production from regional wells located nearest recent earthquake activity (Supplementary Fig. 5). Injection pressure is updated monthly in the model, production data is only updated annually at best due to the limited data available. Injection volumes for Well #1 and Well #2 discussed in the main text are shown in Supplementary Fig. 6. We describe in detail how we integrate subsurface pressure injection and production later in the Methods section.

We define the regional stratigraphy, 3D fault geometry, water injection and production rates using publically available well logs, well log interpretations, and production/injection data provided by the TRC, regional published fault maps and discussions with oil and gas companies operating in the area^20^,^21^,^22^. The match between earthquake epicentre locations and the fault maps generated from well logs ties suggests our proposed fault locations are accurate to within 1 km. The 1-km accuracy of the fault model is limited by uncertainty in the earthquake locations; uncertainty in depth interpolation between well logs used to constrain the fault location, and uncertainties that likely exist in some of the fault interpretations themselves that were supplied by other academic publications and industry researchers. Regional seismic and well log data indicate the Ellenburger is approximately flat-lying with an average thickness of ∼1,000 m (refs 20, 21, 22).

We determine the effective permeability, *k*, for the Ellenburger at this site directly by using the Cooper–Jacob straight-line method that solves for permeability in a single well assuming non-equilibrium radial flow in a confined aquifer^36^:


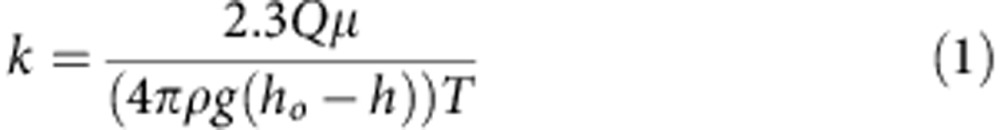


Where *k* is permeability in m^2^, *Q* is the pump rate in m^3^ per day, (*h*_*o*_−*h*) is the drawdown in head per log cycle of time in metres, *ρ* is the fluid density that we set at 1,031 kg m^−3^, *g* is the gravitational acceleration constant, *μ* is the fluid viscosity, set at 1.1 × 10^−3^ Pa s, and *T* is the thickness of the Ellenburger where high permeability exist, which we vary between 300 and 1,000 m. Pump rates and drawdown in pressure for injector well #1 were provided by XTO Energy Inc. and are shown in Supplementary Fig. 8.

Using these data, we estimate the average effective permeability in the injection interval of the Ellenburger near injector well #1 ranges between 3 × 10^−15^ and 1 × 10^−13^ m^2^. We assign a permeability of 1 × 10^−18^ m^2^ to the overlying Barnett shale and underlying granite^37^. Faults sometimes form seals in petroleum reservoirs and can in some instance act as strong barriers to cross fault flow^24^,^28^,^32^,^38^. We therefore also assign a permeability that is 50% lower (1.5 × 10^−15^–0.5 × 10^−13^ m^2^) than the surrounding Ellenburger formation at the fault. Studies have demonstrated that the permeability of limestone faults is highly complex. Nonetheless, detailed permeability studies of normal faults in limestone host rock indicate a fault core with lower permeability surrounded by a higher permeability damage zone^24^,^28^. This implies higher fluid flow immediately adjacent to the fault, but lower flow across the system.

The 3D model solves the groundwater-flow equation for pressure assuming single-phase flow and nearly flat-lying sedimentary layers, where


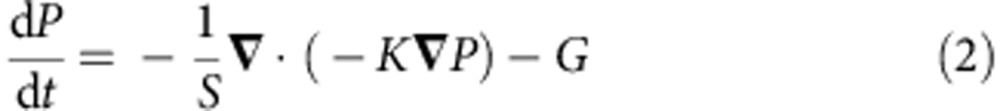


and 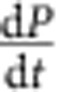 is the change in pressure at a given location with time. *S*, the specific storage, we calculate assuming a mean Ellenburger porosity of 5±3%, a brine compressibility of 4.6 × 10^−10^±0.3 × 10^−10^ Pa^−1^ and a mean rock matrix pore space compressibility for dolomitic limestone of 7 × 10^−10^±6 × 10^−10^ Pa^−1^. The resulting end-member *S* values range from 1 × 10^−6^ m^−1^ to 13 × 10^−6^ m^−1^, with a mean value of 7.3 × 10^−6^ m^−1^. *K*, the hydraulic conductivity, is based directly on previously derived permeability values. **∇***P* is the change in pressure with respect to space and *G* represents potential source (injection well) and sink (producer) terms at a given position and time. We recognize that significant variability in *S* likely exists, and this model therefore only represents a first-order estimate of subsurface pressure.

The 3D numerical model (a derivation of MODFLOW) uses a standard finite-difference forward-time, centre-space explicit approach to model pore pressure evolution with time^22^. The model consists of 194 cells in the north–south direction, 242 cells in the east–west direction and 40 cells in the vertical direction, with cell dimensions of ∼50 × 50 × 50 m. We define the injection interval for injector well #1 and injector well #2 from 2,400 to 2,700 m and 2,200 to 2,850 m, respectively, consistent with reported injection intervals.

Since the shape, size and length of the well tubing and the injection volumes with time are known (made available through the TRC), we use the Darcy–Weisbach equation to calculate potential pressure loss due to friction in the pipe for each well, and from this, calculate the average bottom-hole pressure each month at the injection well site. The Darcy–Weisbach equation is the following:


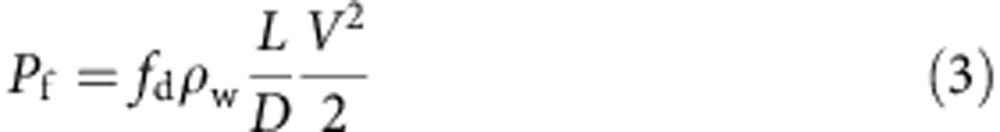


Where *P*_f_ is the pressure loss due to friction, *F*_d_=∼0.02 is the Darcy friction coefficient that is calculated directly using the Colebrook approximation for smooth oilfield pipe tubing^39^, *ρ*_w_ =1,031 is the density of the injected fluid, *L* is the length of the pipe tubing to the packer, *D* is the internal diameter of the pipe and *V* is the average fluid velocity down the pipe. For injector well #1, *L*=2,427 m, *D*=0.102 m and *V* we estimate equals on average 2.3 m s^−1^ based on mean injector volumes with time and tubing surface area. As an example using this approach, we calculate that for an average wellhead pressure of 4 MPa at injector well #1 the pressure is reduced by ∼1.25 MPa at the bottom of the well, so that the average bottom-hole pressure is 2.75 MPa For injector well #2, *L*=2238, *D*=0.076 m and the average *V* is estimated equal to 1.5 m per s based on mean injection volumes with time and tubing surface area. From these parameters, we calculate that the average wellhead pressure at injector well #2 of 3.61 MPa is reduced by 0.69 MPa at the bottom of injector well #1, so that the average bottom-hole pressure is 2.92 MPa.We apply this technique to the monthly pressure/volume data provided for the well sites to estimate how bottom-hole pressure changes with time for each of the injectors.

Whether the bottom-hole pressure estimates using the Darcy–Weisbach equation are accurate is unclear, as no direct bottom-hole pressure measurements exist during pumping and solutions to the equation depend on several time-dependent factors such as flow and friction loss as well as uncertainties in pipe roughness changes. As an alternative approach for estimating bottom-hole pressure, we also estimate the subsurface pressure generated during injection using the Dupuit–Theim equation (the conical solution of Darcy's Law). This approach, unlike the Darcy–Weisbach equation that is primarily empirically based, estimates pressure by conserving mass and momentum, and has the following form:


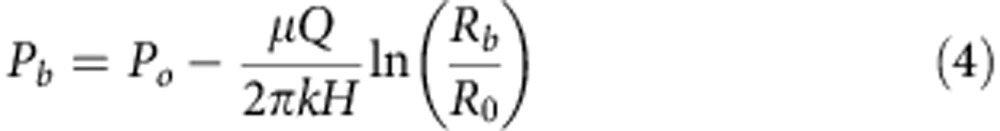


where, *P*_*b*_ is the pressure above hydrostatic at the base of the well, *P*_*o*_=0 is the pressure above hydrostatic at a distance of *Ro*, *μ*=1.1 × 10^−3^ Pa  is the fluid viscosity, *k*=3 × 10^−15^–1 × 10^−13^ m^2^ is the end-member mean effective permeability, *Q* is the average fluid flux out of the injector wells determined from monthly injection values provided by the TRC, *H*=1,000 m is the approximate thickness of the reservoir (a maximum estimate for the pipe perforation zone and therefore minimum bottom-hole pressure estimate), *R*_*b*_=0.1 m is the radius of the production casing and *R*_*o*_=1.5–150 km is the radial distance where no elevated fluid pressure exists with the maximum value defined by the approximate radial distance of the Fort Worth Basin and the minimum value representing the nearest distance to the fault. On the basis of parameter uncertainties listed above and possible uncertainties in bottom-hole location of 50 m, we estimate end-member monthly injector well #1 bottom-hole pressures above *in situ* range from 0.53 to 20 MPa and end-member monthly injector well #2 bottom-hole pressures range from 0.17 to 8 MPa. Permeability plays an important role in the estimation of bottom-hole pressure using this method, and only in cases of low permeability (∼<3 × 10^−13^ m^2^) do high injection pressures (∼>8 MPa) develop in the model. For our analysis, we only focus our results on more realistic, higher permeability values, where bottom-hole pressures are consistently below reported wellhead pressures (Table 1). As noted in Table 1, even in the conservative instance where bottom hole excess pressures are at a minimum 0.07–0.34 MPa, the pressure development along and near the fault is an order of magnitude greater than the stress change associated with lake level or groundwater change.

As noted previously, oil and gas production in the Fort Worth Basin involves not only the injection but the removal of brine, which we model as being entirely from the Ellenburger. Geophysicists working at production companies in the Fort Worth Basin indicate that brine is sometimes produced in the Ellenburger when an occasional frack-job fractures into a fault, or fractures through the Barnett into the Ellenburger formation, especially in regions where the Viola shale is absent below the Barnett shale^20^,^37^—the case for the area were recent seismicity has occurred near Azle. To account for potential pressure reductions caused by Ellenburger production, the model incorporates pressure sinks generated by the production (and removal) of brine from the Ellenburger formation. For this analysis, we assume that potential production from the Ellenburger extends into fractures up to 500 m below the Barnett Shale, to a depth of ∼2,500 m. This is an arbitrary depth estimate for fracture extension into the Ellenburger and in reality these fractures could be shallower or deeper. Currently, we do not know how continuous fractures are in the Ellenburger, although regional seismic images suggest natural fractures could extend through the entire Ellenburger and into the Barnett shale^21^,^22^. Importantly, even if the water is produced only in the upper few metres of the Ellenburger, the change in pressure caused by water extraction will still impact other areas of the Ellenburger formation due to the nature of pore pressure diffusion. For brine production numbers, we use values for the region based on brine production reports made publically available through the TRC from 70 regional wells near the NEFZ that have the largest water production in the region (Supplementary Table 5; Supplementary Fig. 5). Production data are provided in both G-1 and G-10 reports at the TRC. G-1 reports indicate the brine production during the first 48 h of production at a new well and therefore likely over-estimate long-term water production at a site since significant amounts of frack-water can be produced. G-10 reports represent a potentially more accurate estimate of brine production. Unfortunately, G-10 reports, like G-1 reports, are report as only 48-h pump test results that are conducted at most only on an annual basis. Thus, G-1 and G-10 reports represent only the gross estimates for regional brine production at each site. Although brine production often tracks with gas production, the lack of temporal resolution for brine production data makes it difficult to determine with high temporal resolution a clear time correlation between fluid production and seismicity at this site. For simplicity, we present only an average brine production value for each well in the region and typically discard G-1 reports, where anomalously high water production is observed. Future models will include more detailed brine production values if such data are made available.

For our analysis, we assume all brine produced from surrounding oil and gas wells near the NEFZ system derive from the Ellenburger formation. In reality, some of this brine could also derive from the Marble Falls formation, the release of interstitial formation brine from the Barnett or is water originally used for hydraulic fracturing. The estimated monthly water produced from individual production wells near the NEFZ yield volumes that are generally one to two orders of magnitude lower than wastewater injection well injection volumes. However, the sum of all water produced in surrounding wells during any given time could be as much as 35% of waste water injection volumes, assuming extrapolation of brine production estimates available at the TRC are accurate.

The model run time is for a 10-year period (from 2004 until the end of 2013). Model results demonstrate that end-member excess pressures of 0.008–0.2 MPa develop across the faults in areas where earthquake activity exists depending on model parameters. We find that higher pressures form along the fault for model runs where the Ellenburger contains laterally a continuous high-permeability zone bounded by lower permeability rock and a lower permeability fault. To test the role of the faults, we also ran the model with no faults. For this, we observe excess pressures in the earthquake region that are usually within 50–90% of values observed for model runs where faults exists (Supplementary Fig. 9). This implies the location and volume/pressures of injectors and producers are more important factors defining the subsurface pressure regime than the current fault permeability values prescribed in the model.

### Modelling uncertainties

*Compressibility and specific storage uncertainties*. Although we vary compressibility and reservoir-specific storage for different model runs, the compressibility/specific storage for each model run is held constant throughout. Variations in calculated specific storage may change by an order of magnitude, and we find that this uncertainty may result in a 10–15% change in pressure along the fault (Table 1). Future work should base compressibility/specific storage on actual measurements for porous Ellenburger from the region, if available.

### Permeability uncertainties

Although we vary the permeability of the Ellenburger by up to three orders of magnitude, each individual reservoir model assumes a mean effective permeability that is isotropic in different geological units except at the fault locations. In reality, it is likely that significant anisotropy may exist due to orientation of fractures in the subsurface. Future models should account for the orientation and magnitude of permeability anisotropy in the Ellenburger formation and surrounding faults/units once such data become available. Conversations with industry experts indicate that tremendous heterogeneity exists in the Ellenburger over short (<m) depth intervals. 3D seismic analysis of the Ellenburger indicates significant heterogeneity exists along polygonal fracture zones^20^,^21^; fluid flow along higher permeability polygonal fractures could result in high pore-pressure development along the NEFZ relatively rapidly, since the flow would be channelled and more focused than the model suggests. Model runs where we supply a thinner zone (300 m or less) of high and more focused permeability material in the Ellenburger (which might represent a karst-like feature that exist in this formation) results in significant pressure changes at the fault, with pressures as much as a factor of 4 higher. Currently, the mean effective thickness of the flow zone in the Ellenburger is poorly constrained at this site, and as a conservative approach, we assume it is isotropic and thick, with uniform permeability throughout the entire 1,000 m Ellenburger formation. Tracer tests provide one valuable approach to constrain flow path, effective permeability and Ellenburger production rates at producer wells, and such tests should be considered in the future.

In addition, a less expensive, yet valuable approach for assessing effective permeability as well as the potential for induced seismicity due to oil and gas activities is through 24 h shut-in tests at injector wells. Such shut-in periods can be used both to estimate regional permeability near each injector well via the Cooper–Jacob Method and to determine if background *in situ* bottom-hole pressures are changing significantly with time. Annual 24-h shut-in tests or required pressure measurements during shut-in for maintenance would provide potentially critical insight into wastewater reservoir pressure changes with time that may lead to induced seismicity.

### Brine production uncertainties

As previously noted, significant brine production uncertainty exists in the model. It is critical that future studies include high-resolution (ideally daily) brine production data for producing wells and better constraints on the source of brine production. Currently, all brine production data included in the model are based on extrapolations and averages of G-10 forms provided by the TRC, which are based only on 48-hour pump tests that are typically performed annually. Comparison of different annual G-10 reports for the same well indicates brine production can vary significantly from year to year, depending on the well. As a result, pressure changes associated with modelled brine production represent only a crude, first-order estimate. The depth/location of brine production is also limited to a resolution of a few hundred metres due to hydraulic fracturing zones extending sometimes over hundreds of metres. This uncertainty, however, is currently significantly less important than better constraints on brine production volumes with time and the brine source. Tracer tests or geochemical studies determining if the chlorinity content of the brine produced matches Ellenburger values would significantly help constrain brine source uncertainties.

### Bottom-hole pressure uncertainties

Although we calculate bottom-hole pressures by incorporating pressure losses due to friction in the tubing and conservation of mass/momentum, it is unclear if these calculated pressure losses accurately reflect true bottom-hole pressures. More advanced pump tests including low-cost shut-in pressure measurements, and ideally, bottom-hole pressure measurement at injector wells and nearby sites can further elucidate pressure loss and true bottom-hole pressure. In addition, the model does not account for non-Darcy flow that likely occurs in the formation nearest production and injection well bores, and future models should consider the likely impact of such effects.

### Regional structural geology uncertainties

Interpretations of region well logs made publically available by the TRC provide first-order insight into regional structural geology. Nonetheless, access to 3D seismic data and 3D structural interpretations based on high-resolution 3D seismic data are necessary to make the most accurate pore pressure model for the region. Although we note that two faults exist in the region, discussions with industry researchers indicate several large karst features also exist in this region. Some, but not all of these, features are observable in 3D seismic data and it is likely that these features represent zones of significant permeability changes. Seismic interpretations provided by industry researchers have been an invaluable tool for constrain regional structure. Access to 3D seismic data, or access to interpretations of such data, would therefore provide greater insight into the complex potential flow paths that exist in the subsurface.

### Stress magnitude and orientation uncertainty

Improving the certainty of whether pressure changes associated with oil and gas activity are the primary cause of earthquakes requires a more detailed understanding of the subsurface stress regime that defines not only the orientation of the stress field, but also quantifies the stress changes necessary to cause failure. Detailed analysis of regional subsurface stress combined with longer-term regional stress studies will likely provide invaluable insight into the regional stress regime and the potential stresses required to induce failure on faults in this region.

## Author contributions

M.J.H., W.L.E., C.F., J.E.O. and C.B. developed/refined pore pressure and stress models. H.R.D., H.R.O., C.H., W.L.E., B.W.S., M.B.M. and J.H.L. developed/deployed the seismic experiment and processed/interpreted all seismic data. All authors shared in the writing of this manuscript.

## Additional information

**How to cite this article:** Hornbach, M. J. *et al.* Causal factors for seismicity near Azle, Texas. *Nat. Commun.* 6:6728 doi: 10.1038/ncomms7728 (2015).

## Supplementary Material

Supplementary Information Supplementary Figures 1-9, Supplementary Tables 1-5, Supplementary Notes 1-4 and Supplementary References

## Figures and Tables

**Figure 1:**
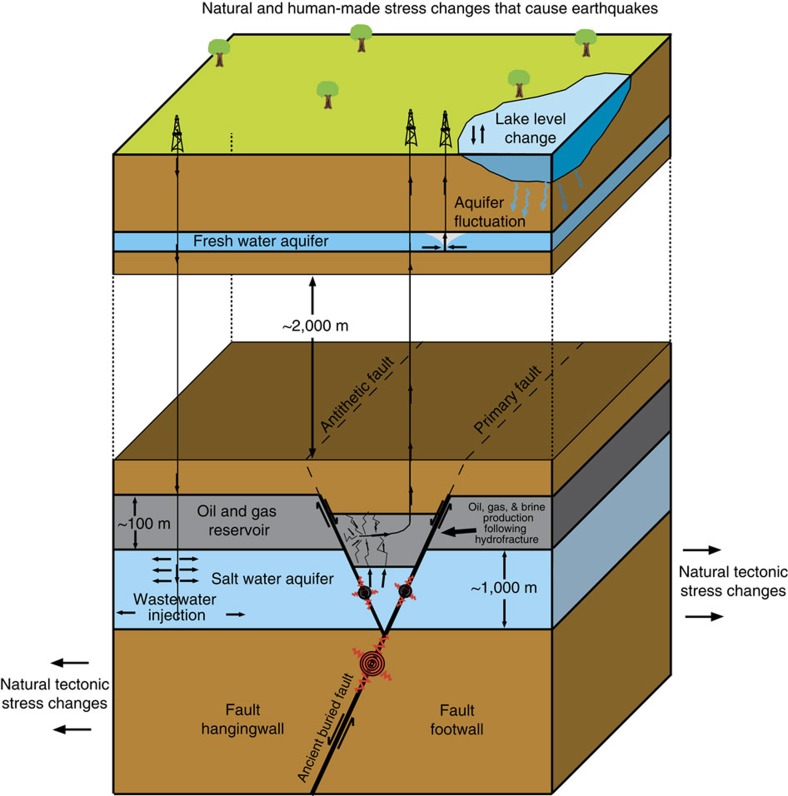
Natural and anthropogenic stress changes that may trigger earthquakes in the Azle area. Several natural and anthropogenic (man-made) factors can influence the subsurface stress regime resulting in earthquakes. Natural stress changes that promote earthquakes include intraplate stress changes related to plate tectonics^9^,^10^ and natural water table or lake levels variations caused by changing weather patterns or water drainage patterns with time, and in some instances (not pictured) the advance or retreat of glaciers. Anthropogenic stress changes that promote earthquakes include human-generated changes to the water table (including dam construction^2^,^3^) and industrial activities involving the injection or removal of fluids from the subsurface^4^. The figure is not to scale.

**Table 1:** Examples of model parameters and associated results.

**Well #1 mean excess bottom-hole pressure in (MPa)**	**Well #2 mean excess bottom-hole pressure in (MPa)**	**Mean effective permeability ( × 10**^**−14**^ **m**^**2**^**)**	**Thickness of high permeability zone (m)**	**Producers included?**	**Boundary conditions**	**Specific storage ( × 10**^**−6**^ **m**^−**1**^**)**	**Excess pressure on fault at AZDA, 1 January 2014 (MPa)**
0.53	0.17	3	1,000	Yes	Closed	5	0.008
0.53	0.17	3	1,000	Yes	Closed	13	0.02
0.53	0.17	3	1,000	No	Closed	7.3	0.011
4.4	2.96	3	300	No	Closed	7.3	0.14
2.42	1.63	3	300	No	Closed	7.3	0.08
2.42	1.63	3	300	No	Open	7.3	0.015
2.42	1.63	3	1,000	Yes	Closed	13	0.03
2.42	1.63	3	1,000	No	Closed	5	0.05
2.42	1.63	3	1,000	No	Open	5	0.01
2.42	1.63	1	1,000	Yes	Closed	1	0.11
2.42	1.63	1	1,000	Yes	Closed	13	0.1
2.42	1.63	1	1,000	Yes	Closed	7.3	0.11
0.58	0.28	5	1,000	Yes	Open	7.3	0.02
2.42	1.63	5	1,000	Yes	Closed	7.3	0.1
2.42	1.63	10	1,000	Yes	Open	7.3	0.017
