# Supplementary material for: Causal factors for seismicity near Azle, Texas
Source: Nat Commun. 2015 Apr 21;6:6728. doi: 10.1038/ncomms7728 (PMC4410634; doi:10.1038/ncomms7728)
Supplement: Supplementary Information — Supplementary Figures 1-9, Supplementary Tables 1-5, Supplementary Notes 1-4 and Supplementary References [file ncomms7728-s1.pdf]

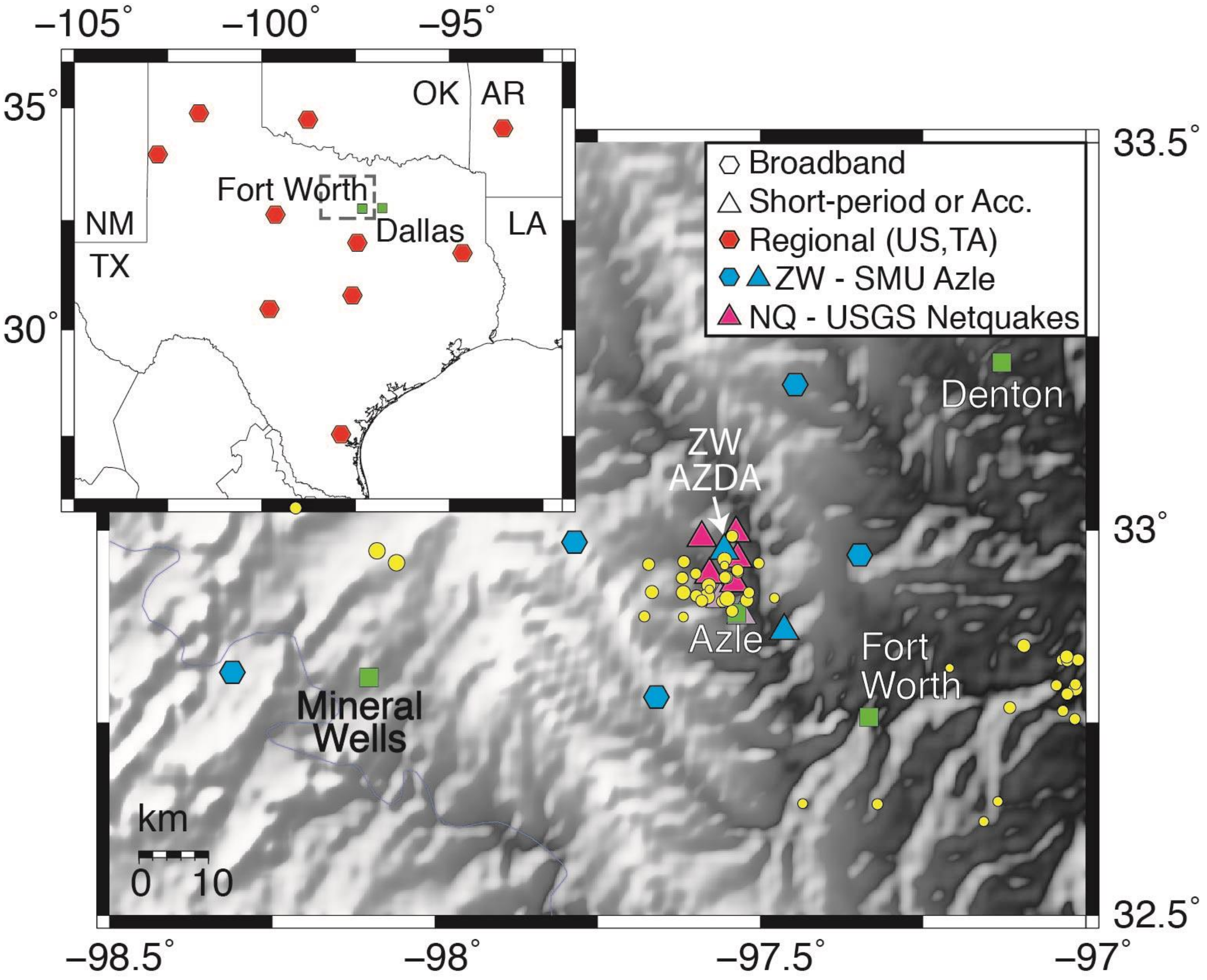

Supplementary Figure 1. USGS NEIC catalogue locations 2008-present (yellow circles) and seismograph stations for North Texas. The Azle earthquake sequence consists of the 27 felt events located to the north and west of Azle, TX (shown here) and seismicity recorded by the temporary network (see Figure 1). Additionally, two M3.7 earthquakes near Mineral Wells occurred in November 2013. NEIC locations are determined using the regional broadband stations (red hexagons) shown in the inset. The temporary Azle network (ZW and 5 NQ stations) was deployed starting on 15 December 2013 and completed to 12 stations by mid-January 2014.

:

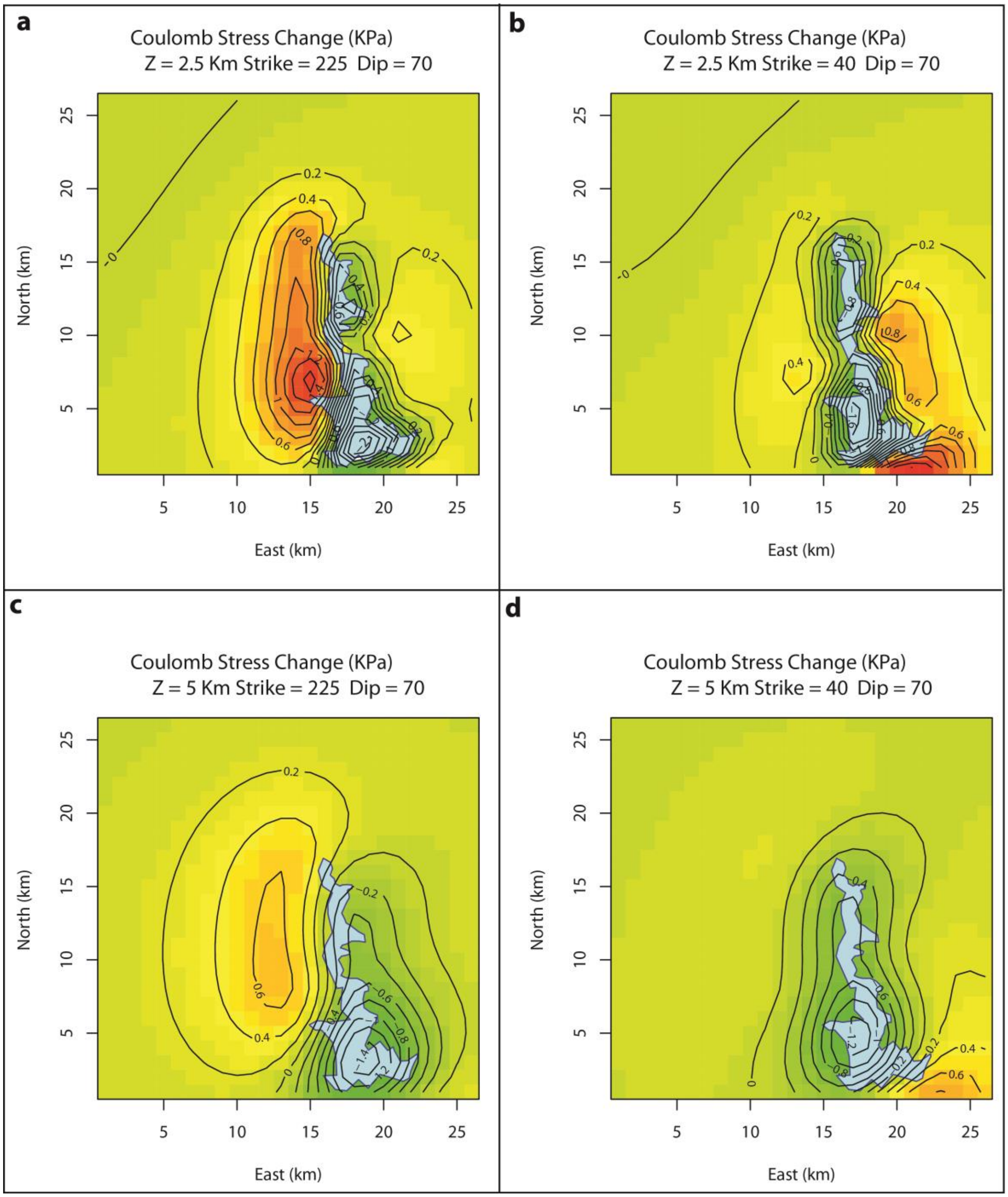

Supplementary Figure 2. Change in Coulomb stress at 2.5 km (**a**, **b**) and 5 km (**c**, **d**) depth for normal faulting caused by the 2.1 m drop in water level in Eagle Mountain Lake between April 2012 and November 2013, computed using the Boussinesq solution for a change in surface load on an elastic half-space[37]. Warm colours indicate increased failure potential; cool colours indicate decreased failure potential. **a** and **c** correspond to orientation of the main fault defined by earthquake hypocentres. **b** and **d** correspond to antithetic normal fault orientation.

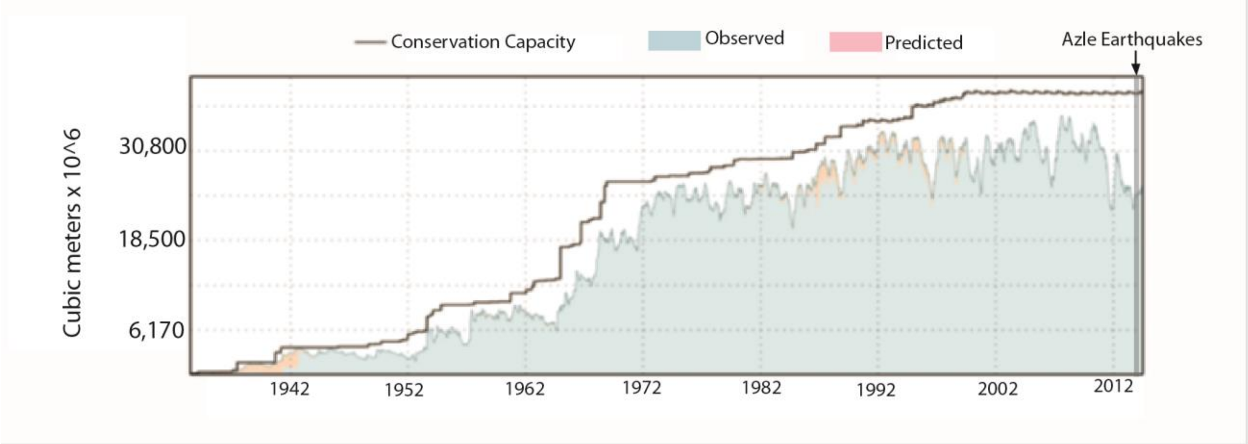

Supplementary Figure 3. Water volume stored in Eagle Mountain Lake since dam construction in 1932 (http://www.waterdatafortexas.org/reservoirs/statewide). During the period of earthquake activity, lake volumes have not been at record high or record low values.

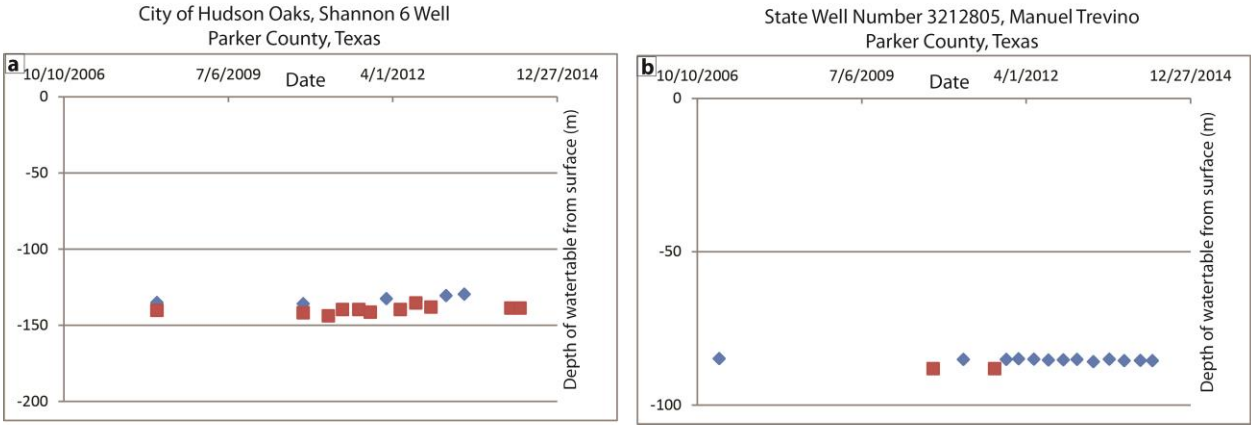

Supplementary Figure 4. Depth from the surface to the top of the unconfined Trinity Aquifer at two wells near Azle, Texas, in metres, monitored and provided by the Upper Trinity Groundwater Conservation District. Unfortunately, no Trinity Aquifer monitoring wells exist directly over the earthquake area. The Shannon #6 Well (**a**) is located at 32.7479 N, -97.7032 W, and appears to be the closest Trinity Aquifer Monitoring Well to the earthquakes. The Manuel Trevino Well (**b**), located 32.783 N, -97.573 W, provides another example of water levels in the Trinity Aquifer in Parker County. Blue diamonds are static water levels; red squares are pumping water levels. Water levels of the Trinity aquifer appear to fluctuate vertically by a few metres on an annual basis, and therefore likely have a similar negligible impact on the subsurface stress regime as lake level change at Eagle Lake Reservoir.

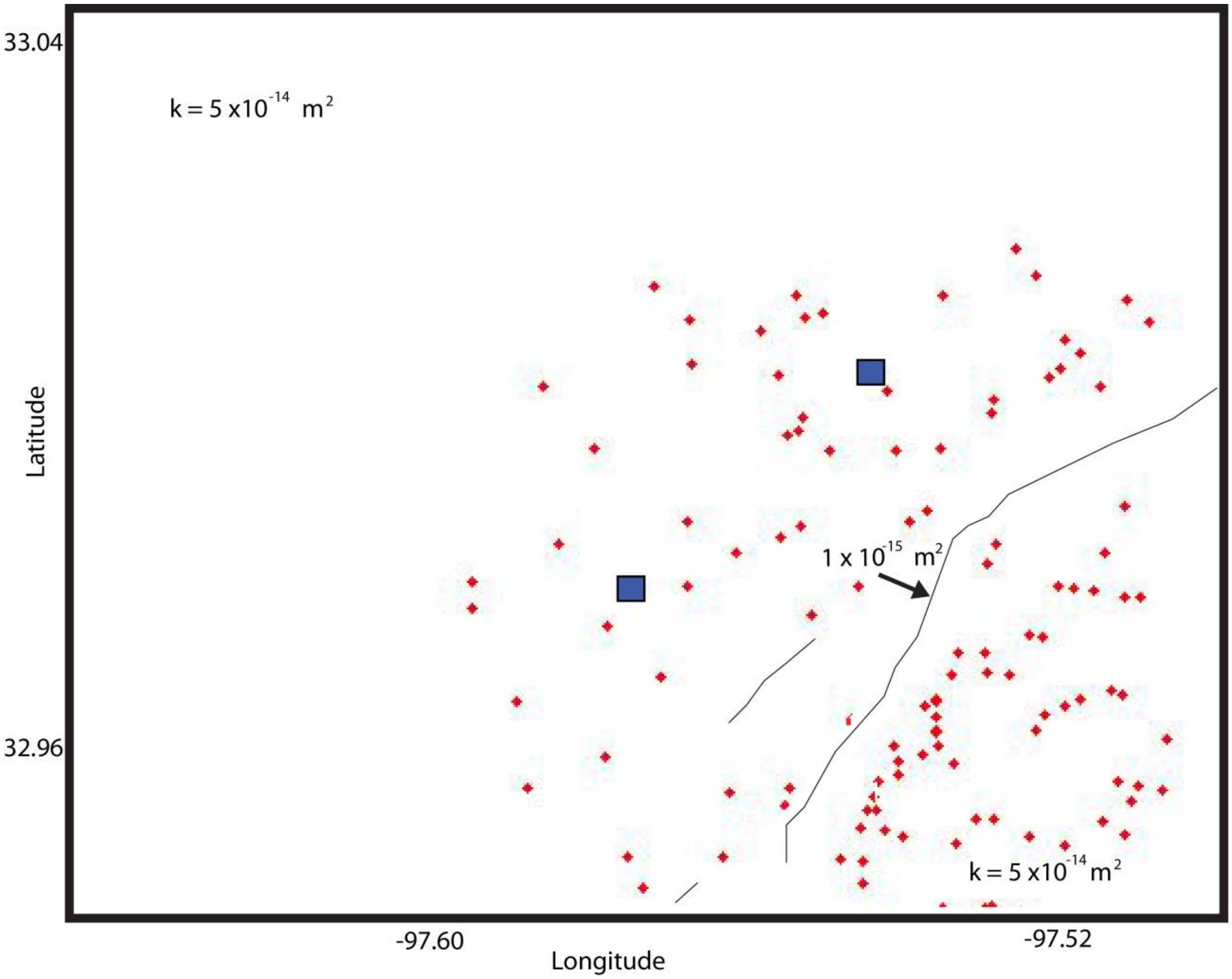

Supplementary Figure 5. Map view of model space at a depth of 2500 m showing the location of the two injector wells (blue), the 70 largest brine producer wells in the region (red), and an example of assigned mean effective permeability values for the Ellenburger formation versus fault permeability for an example model run. Black lines indicate the fault location at the top of the Ellenburger formation.

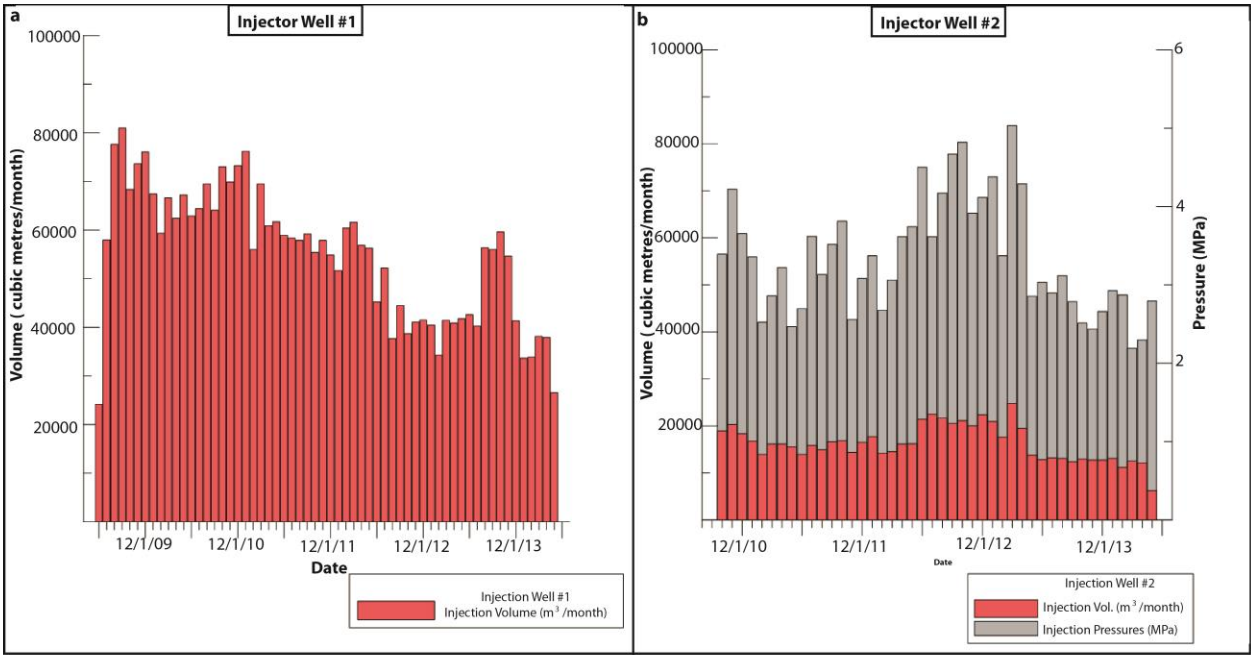

Supplementary Figure 6. Monthly Injection volumes up to September, 2013 for Injector Well #1 (**a**) and Injector Well #2 (**b**) based on TRC public data. See also Supplementary Tables 3 and 4. Grey bars indicate the average monthly wellhead pressure reported for Injector Well #2. Note that average pressure values generally increase and decrease with increasing and decreasing injection volumes. Nonetheless, during the period of earthquake activity (~12/1/13), the injection pressure increases without a clear increase in injection volume implying decreasing compressibility in the injection reservoir during the period of earthquake activity. Injector Well #1 owners provide average daily injection pressures and volumes below (Supplementary Figure 7).

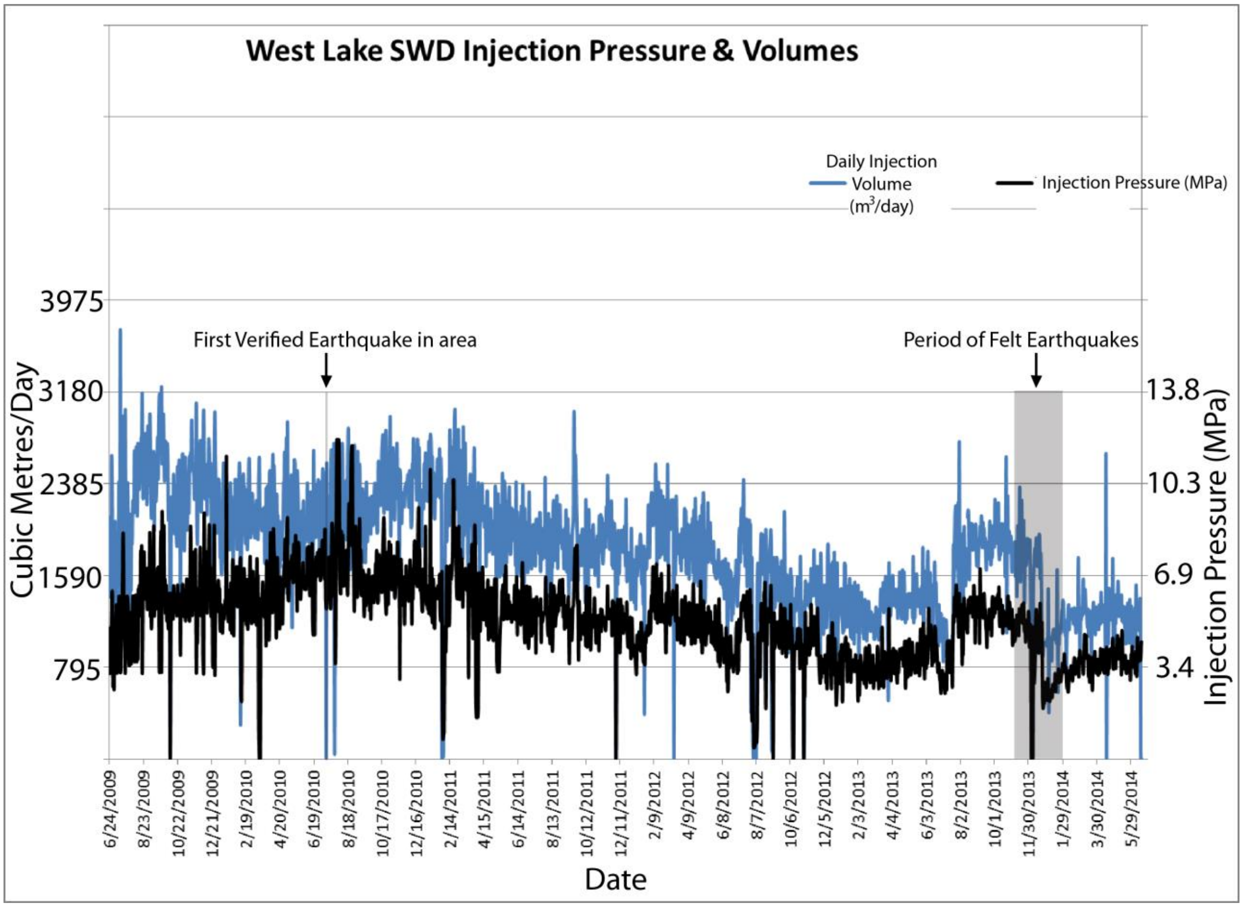

Supplementary Figure 7. Average daily injection pressures for Injector Well #1 since the start of injection provided by XTO Energy Inc. and now available through the TRC. Although a pressure increase occurs in late 2013, three months before felt earthquake activity begins, it is also clear that injection well pressures and volumes were significantly higher during the 2009-2011 period when only one recorded earthquake was reported by a temporary seismic network was placed in the region from November 2009 to September 2011 [17]. Data provided courtesy of XTO Energy Inc.

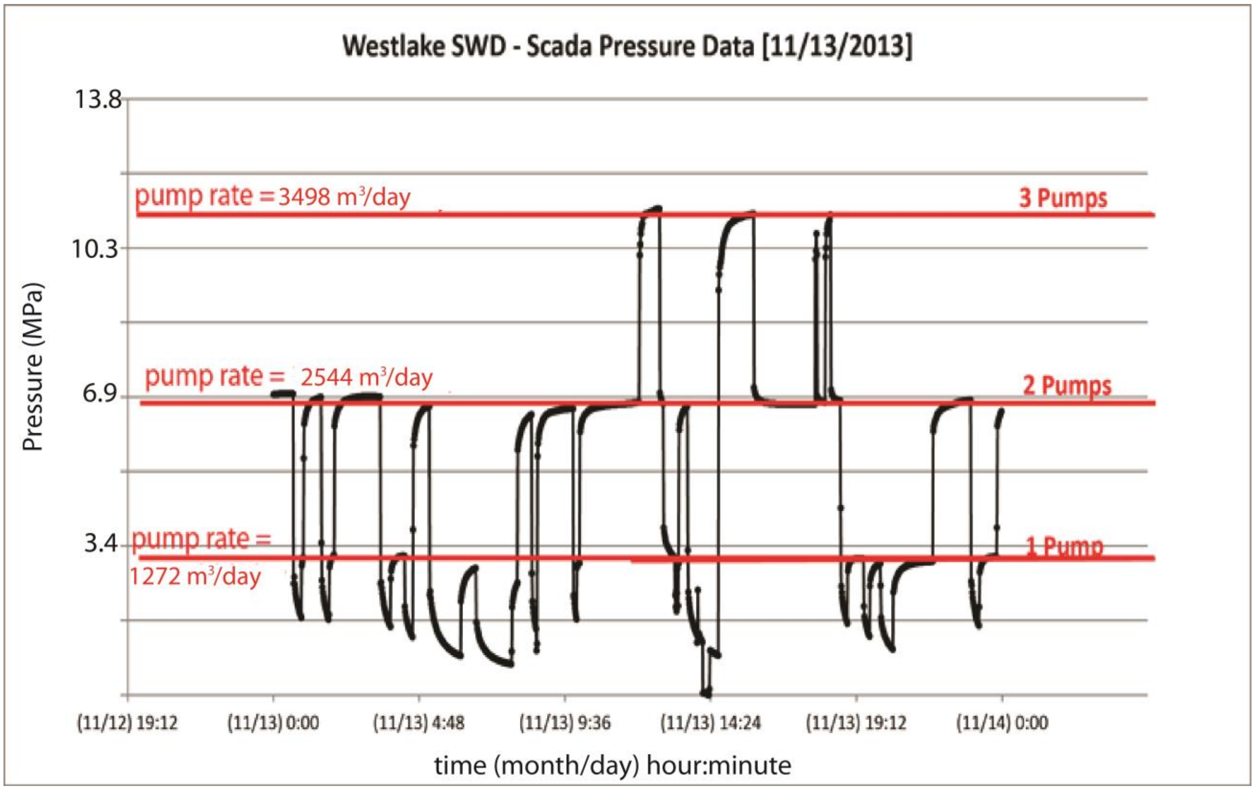

Supplementary Figure 8. Measured wellhead pressures at Injector Well #1 for time periods where different pump rates and periods of no pumping occurred. We use these data to place constraints on permeability in the Ellenburger near Injector Well #1. During periods when all injection pumps are off, wellhead pressure exponentially decays. Industry researchers indicate that during periods of sustained shutdown (several days) well head pressures converge to ~0.5 MPa. Data provided courtesy of XTO Energy Inc.

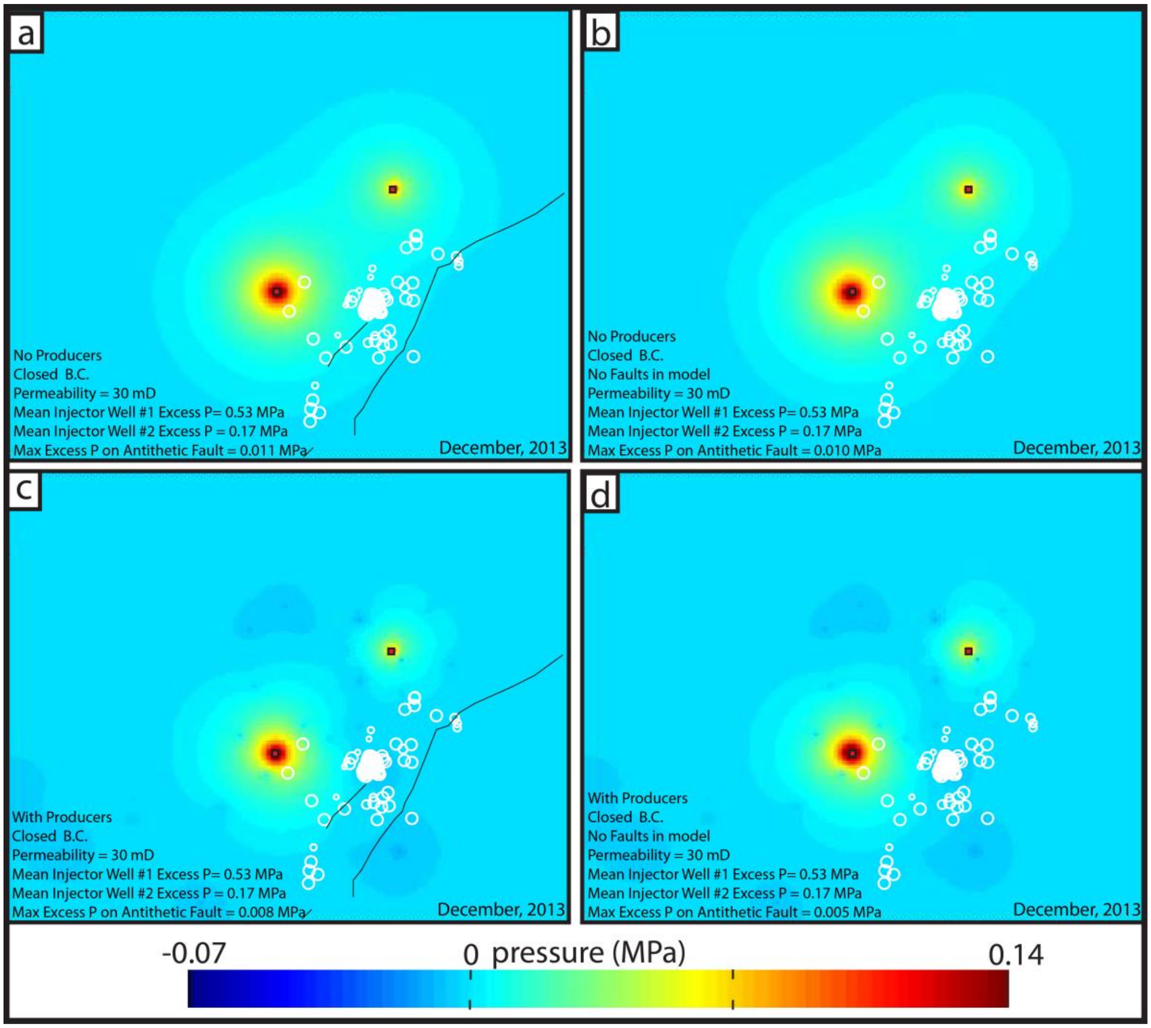

Supplementary Figure 9. Estimate for excess pressure in the Ellenburger, December 2013, based on model results assuming average pressures of 0.57 MPa and 0.17 MPa exist at Injector Well #1 and Injector Well #2, respectively. These injection pressures are low end-member estimates. For all models, the Ellenburger is 1000 m thick. **(a)** only brine injection occurs; **(b)** only brine injection occurs and no subsurface faults exist; **(c)** brine injection and water production occur, and **(d)** brine injection and water production occur and no faults exist. The existence of faults and no production wells results in the largest pressure development at earthquake locations. The scenario with no faults and brine production results in the lowest pressure development in the area of earthquakes locations. Even for the lowest pressure case, model-predicted pressure is still ~1 order of magnitude higher than the expected pressure changes caused by lake level and ground water changes near the surface.

Supplementary Table 1:

Seismic Station information

Station	Network	Ondate*	Offdate*	Latitude	Longitude	Elevation (km)	Instrument	Sample Rate (samples per second)
AZCT	NQ	2013348	2014007	32.9103	-97.5617	0.223	GeoSIG GMS-IA18 NetQuakes	200
AZEP	NQ	2014007	-	32.9634	-97.5354	0.224	GeoSIG GMS-IA18 NetQuakes	200
AZFS	NQ	2013346	2013358	32.8891	-97.5291	0.135	GeoSIG GMS-IA18 NetQuakes	200
AZHS	NQ	2013348	-	32.9297	-97.5397	0.219	GeoSIG GMS-IA18 NetQuakes	200
AZNH	NQ	2013358	-	32.989	-97.5904	0.273	GeoSIG GMS-IA18 NetQuakes	200
AZCF	ZW	2014030	-	33.1841	-97.4463	0.381	Guralp CMG3T_120sec/Guralp DM24 Datalogger	100
AZDA	ZW	2014008	-	32.9728	-97.5553	0.238	Mark Products L28/Reftek 130 Datalogger	100
AZDA	ZW	2014008	-	32.9728	-97.5553	0.238	Chapparral 2.5 microphone/Reftek 130 Datalogger	40/200
AZDA	ZW	2014008	-	32.9728	-97.5553	0.238	Mark Products L4C/Reftek 130 Datalogger	100/200
AZHL	ZW	2014011	-	32.9656	-97.3483	0.222	Guralp CMG3T_120sec/Guralp DM24 Datalogger	100
AZLE	ZW	2014011	-	32.9824	-97.7862	0.381	Guralp CMG3T_120sec/Guralp DM24 Datalogger	100
AZWP	ZW	2014015	-	32.7795	-97.66	0.381	Guralp CMG3T_120sec/Guralp DM24 Datalogger	100
AZWR	ZW	2014028	-	32.8115	-98.312	0.381	Guralp CMG3T_120sec/Guralp DM24 Datalogger	100
BRRD	NQ	2013354	-	32.9948	-97.5379	0.223	GeoSIG GMS-IA18 NetQuakes	200
EML1	ZW	2013348	-	32.8735	-97.4603	0.211	Sprengnether S6000/Reftek 130 Datalogger	100
RESD	NQ	2013348	-	32.9419	-97.5786	0.223	GeoSIG GMS-IA18 NetQuakes	200

*Ondate and Offdate in YearJulianDay

Supplementary Table 2:

Local P-wave (VP) and S-wave (VS) Velocity Model Used to Relocate Earthquakes Near Azle, Texas.

Depth BSL (km)*	Depth Surface (km)	VP (km s^{-1})	VS (km s^{-1})	VP/VS
-0.235	0.000	2.75	1.53	1.8
0.135	0.370	3.70	2.05	1.8
0.565	0.800	4.00	2.22	1.8
1.465	1.700	4.35	2.44	1.8
1.865	2.100	6.00	3.37	1.8

Supplementary Table 3:

Monthly Injection Rates for Injection Well #1

Time (month-year)	m^3 per month
Jun-09	18135
Jul-09	43451
Aug-09	58222
Sep-09	60730
Oct-09	51254
Nov-09	55239
Dec-09	57047
Jan-10	50566
Feb-10	44490
Mar-10	49979
Apr-10	46830
May-10	50399
Jun-10	47146
Jul-10	48299
Aug-10	52117
Sep-10	48041
Oct-10	54769
Nov-10	52380
Dec-10	54903
Jan-11	57145
Feb-11	41982
Mar-11	52118
Apr-11	45664
May-11	46272
Jun-11	44180
Jul-11	43758
Aug-11	43391
Sep-11	44397

Oct-11	41548
Nov-11	43401
Dec-11	41127
Jan-12	38728
Feb-12	45290
Mar-12	46196
Apr-12	42636
May-12	42186
Jun-12	33910
Jul-12	39147
Aug-12	28259
Sep-12	33347
Oct-12	29015
Nov-12	30802
Dec-12	31106
Jan-13	30343
Feb-13	25720
Mar-13	31080
Apr-13	30640
May-13	31295
Jun-13	31927
Jul-13	30170
Aug-13	42264
Sep-13	41987
**Total**	**2225028**
**Mean**	**43628**

Supplementary Table 4:

Monthly injection rates and average wellhead pressure rates for Injector Well #2.

Time (month-year)	Av. Monthly Pressure (MPa)	Volume (m^3 per month)
Oct-10	3.4	14194
Nov-10	4.2	15207
Dec-10	3.7	13746
Jan-11	3.4	12580
Feb-11	2.5	10419
Mar-11	2.9	12123
Apr-11	3.2	12136
May-11	2.5	11658
Jun-11	2.7	10427
Jul-11	3.6	11888
Aug-11	3.1	11223
Sep-11	3.5	12488
Oct-11	3.8	12647
Nov-11	2.6	10778
Dec-11	3.1	12378
Jan-12	3.4	13294
Feb-12	2.7	10649
Mar-12	3.1	10870
Apr-12	3.6	12116
May-12	3.7	12153
Jun-12	4.5	16055
Jul-12	3.6	16875
Aug-12	4.2	16264
Sep-12	4.7	15381

Oct-12	4.8	15843
Nov-12	3.9	15010
Dec-12	4.1	16784
Jan-13	4.4	15710
Feb-13	3.4	13220
Mar-13	5.0	18584
Apr-13	4.3	14582
May-13	2.9	10342
Jun-13	3.0	9603
Jul-13	2.9	9918
Aug-13	3.1	9792
Sep-13	2.8	9275
**Total**		**466213**

Supplementary Table 5:

Production well number (American Petroleum Institute "API" number), location, and start date for the 70 largest brine-producing production wells in the earthquake area based on G-10 TRC reports. All G-10 data can be found through the TRC.

API	Latitude	Longitude	Mean Monthly brine production (m^3 per month)	Start Date
43932613	32.97092	-97.5304	2770	Oct. 2008
36735205	32.9562	-97.5898	1297	Jan. 2012
43932605	32.97099	-97.534	1161	Oct. 2008
43935738	32.94141	-97.5267	1116	Sept. 2011
36733989	32.985277	-97.54019	980	Oct. 2006
36734139	32.96381	-97.5476	683	Nov. 2006
49736104	33.00734	-97.5091	539	Nov. 2006
43935807	32.94141	-97.5267	533	Aug. 2011
36734349	32.95557	-97.5635	522	May, 2007
43934469	32.94124	-97.5285	467	Mar. 2010
36733762	32.95958	-97.5795	448	June, 2006
43931502	32.98667	-97.5378	446	Aug. 2006
43935733	32.94322	-97.5296	417	July, 2011
36733709	32.956184	-97.55569	385	Sept. 2005
36734045	32.97524	-97.5528	370	Oct. 2006
43935846	32.9418	-97.5257	368	Aug. 2011
43931850	32.96596	-97.5179	337	Aug. 2007
36734070	32.984983	-97.55416	329	Sept. 2006
49736435	33.00393	-97.518	306	July, 2008
36735272	32.97873	-97.5967	304	Aug. 2012
43934619	32.97791	-97.5163	295	April, 2010
36735271	32.97589	-97.5969	290	Jan, 2013
49736433	33.00537	-97.52	290	July, 2008
36734715	32.96238	-97.5367	284	Nov. 2008
36733868	32.9659	-97.5367	281	Mar. 2006
43932449	32.95082	-97.5247	279	Oct. 2007
43936094	32.96507	-97.5201	279	Feb. 2012

43931961	32.98087	-97.53	265	Aug. 2007
49736119	33.00978	-97.512	240	Jan. 2007
43931868	32.95649	-97.5107	235	Dec. 2007
43935732	32.94303	-97.5304	214	Sept. 2011
43933173	32.98303	-97.529	200	June, 2008
36734395	32.965689	-97.5912	195	July, 2007
43931370	32.93891	-97.5272	188	Mar. 2006
36733927	32.96827	-97.5723	180	July, 2006
36734714	32.96513	-97.538	175	Nov. 2008
49736184	33.01524	-97.5265	162	Feb. 2007
43934202	32.94977	-97.5199	162	Dec. 2009
49736702	33.00213	-97.5206	154	Jan. 2009
43931807	32.96871	-97.53	152	Sept. 2007
43934611	32.99339	-97.536	149	Aug. 2010
43931809	32.96862	-97.5346	140	April, 2007
43934088	32.95259	-97.515	137	Dec. 2009
43934486	32.97809	-97.5189	137	June, 2010
43936082	32.96678	-97.5139	129	Mar. 2012
36733734	32.99323	-97.5419	128	Nov. 2005
36735139	32.96571	-97.5367	125	Sept. 2011
36734972	32.956926	-97.54423	119	Sept. 2009
49735655	33.00013	-97.5153	116	Jan. 2005
36734249	32.982997	-97.58554	118	Feb.2008
43936073	32.97837	-97.5208	109	Feb. 2012
36733979	32.94865	-97.5766	104	July, 2006
36735138	32.96393	-97.5368	94	Sept. 2011
43931700	32.95886	-97.5344	94	April, 2007
43931357	32.95098	-97.5124	92	Feb.2006
49736415	32.99882	-97.5291	91	Feb.2008
36733925	32.93989	-97.5461	91	Sept. 2006
49737176	33.00028	-97.5877	91	July, 2011
43934499	32.95176	-97.5464	76	June, 2010
36734628	32.95899	-97.5415	75	Dec. 2008

43934368	32.96245	-97.5239	75	Mar. 2012
36733879	32.95753	-97.5416	73	Sept. 2006
36734744	32.97401	-97.5794	72	Nov. 2008
43933657	32.97287	-97.5246	71	Dec. 2008
49736416	33.00748	-97.5685	71	April, 2008
43936121	32.96645	-97.5125	71	Mar. 2012
36734471	32.95423	-97.5564	69	Nov. 2007
36735080	32.95368	-97.5456	65	Dec. 2010
43936232	32.94811	-97.5462	63	April, 2012
49736003	32.99729	-97.5296	63	July, 2006
